# Editorial: New approaches for the discovery of GPCR ligands

**DOI:** 10.3389/fendo.2023.1272700

**Published:** 2023-08-21

**Authors:** Paula Morales, Magdalena M. Scharf, Cory P. Johnson, Antonella Di Pizio, Daniel Hilger

**Affiliations:** ^1^ Instituto de Química Médica, Consejo Superior de Investigaciones Científicas, Madrid, Spain; ^2^ Department of Physiology and Pharmacology, Karolinska Institutet, Stockholm, Sweden; ^3^ Kathryn W. Davis Center for Regenerative Biology and Aging, Mount Desert Island Biological Laboratory, Bar Harbor, ME, United States; ^4^ In Silico Biology & Machine Learning, Leibniz Institute for Food Systems Biology at the Technical University of Munich, Freising, Germany; ^5^ Chemoinformatics and Protein Modelling, Department of Molecular Life Sciences, Technical University of Munich, Freising, Germany; ^6^ Department of Pharmaceutical Chemistry, Philipps-University of Marburg, Marburg, Germany

**Keywords:** GPCR, ligand, modulator, drug discovery, ligand design, new tools, ligand characterization

G protein-coupled receptors (GPCRs) constitute the largest class of transmembrane receptors in the human genome and transduce a wide range of extracellular signals by eliciting diverse intracellular responses. As such, they play critical roles in human (patho-)physiology and represent important drug targets (1). GPCR drug discovery attracts high interest by the pharma industry, as it presents challenges but also promising opportunities. While traditional drug discovery addresses the orthosteric binding site of GPCRs that is targeted by endogenous ligands, recent attention has focused also on topographically distinct allosteric ligand-binding sites, which offer potential therapeutic benefits (2–4). Ligands addressing these sites often show lower adverse side effects due to the higher sequence variation between allosteric pockets of receptor isoforms and less interference with the spatio-temporal signaling pattern of endogenous agonists.

The development of new GPCR ligands targeting distinct ligand-binding sites is still very challenging and progress is rather slow. Therefore, new innovative strategies and tools are needed to design, develop, and pharmacologically characterize novel GPCR ligands with the aim of creating better and more specific drugs.

This Research Topic in Frontiers in Endocrinology provides a collection of articles focused on the discovery and characterization of new ligands and novel tools for GPCR drug discovery.

Two comprehensive reviews highlight the therapeutic potential of GPCR allosterism. While Shen et al. offer structural insights into recently solved allosteric GPCR complexes and an overview on the mechanistic effects of allosteric modulators on GPCR dynamics and signaling, Nguyen et al. focus on the adenosine 1 receptor (A1R) family. This review summarizes the clinical benefits of allosterically targeting A_1_R and analyzes structural activity relationships of reported positive allosteric modulators. Moreover, the authors state their perspective on the future applications of computational advances in the development of A_1_R allosteric ligands as novel therapeutics using artificial intelligence and virtual screening strategies.

Salama et al. provided an overview of the multiple formyl peptide receptor 2 (FPR2)-mediated functions of serum amyloid A (SAA) on chemotaxis, induction of inflammatory modulators, formation of atherosclerotic plaques, allergy, regulation of apoptosis, macrophage polarization, bone formation, pulmonary disease, and cancer. Furthermore, the authors discuss different posttranslational-modified SAA variants, their biological activities, and potential role as allosteric modulators and therapeutic agents directed against the FPR2.

In their research article, Muñoz-López et al. investigated the molecular mechanism underlying the antitumoral activity of resveratrol on HeLa and SH-SY5Y cells. The authors show that resveratrol affects adenosinergic signaling by modulating the surface expression levels of the adenosine receptor subtypes A_1_ and A_2A_. Specifically, they demonstrated a resveratrol-dependent decrease in the surface expression level of the A_2A_ receptor, while the expression level of the A_1_ receptor was increased. Together, with the attenuated ecto-5’-Nucleotidase (CD73) enzymatic activity observed in the resveratrol-treated human cancer cells, the authors suggest that the antitumoral effect of resveratrol on cancer cells follows a common molecular mechanism that includes the adenosine signaling pathway.

Chen and Obal provide a comprehensive overview of various detection methods and labeling techniques used to characterize GPCR signaling and the spatial distribution of receptors in cells. The authors summarize different optical detection methods commonly employed in these studies, followed by an overview of currently available biosensors based on fluorescence and bioluminescence energy transfer (FRET and BRET) and protein complementation assays (PCA). Applications of these biosensors to study GPCR signaling at various levels, including activation, oligomerization, and spatial distribution of GPCRs, receptor-dependent transducer recruitment and activation, second messenger generation, and transcriptional regulation, are presented. The review further highlights the value of human induced pluripotent stem cells (hiPSCs) for studying GPCR signaling and personalized drug discovery.

In their review article, Salas-Estrada et al. summarize metadynamics-based strategies and statistical analyses of simulation data as well as artificial intelligence-based tools to investigate the molecular mechanism of ligand-based modulation of GPCR function. The authors describe several studies on GPCRs in which metadynamics has been employed to predict ligand binding modes, the activation landscape, ligand-specific receptor conformations, and ligand binding and activation kinetics of GPCRs.

Jones and Jones present a novel theoretical model for GPCR signal transduction based on statistical mechanics and information transmission theory. First, the authors provide a detailed description of their mathematical model before they compare model-based predictions with available experimental results for the angiotensin II type 1 and the β_2_-adrenergic receptor. The model provides some observations, including the importance of receptor dephosphorylation for controlling the signal activity, the discontinuous dependency of biased-signaling response on ligand concentration, and the statistical balance of on/off switches at maximum information flow. It will have some applications for GPCR systems biology and, therefore, a potential impact for drug discovery.

Williams et al. describe the development of novel fluorescent peptide agonists for the apelin receptor, an important therapeutic target in cardiovascular disease. The authors demonstrate the applicability of these fluorescent ligands to characterize the subcellular binding, expression and internalization as well as the tissue distribution of the apelin receptor in human renal samples. Furthermore, the authors provide a proof-of-principle for the application of the fluorescent ligands in drug discovery pipelines to develop new therapeutic compounds for the apelin receptor.

In summary, this Research Topic brings together a series of articles on different aspects of ligand-based regulation of GPCR activation and signaling, including the modulation of receptor function by allosteric ligands, endogenous proteins, and naturally occurring compounds. In addition, the contributing authors present various tools for ligand discovery, such as fluorescent ligands, FRET- and BRET-based GPCR signaling reporters, metadynamics-based strategies and a novel theoretical model of GPCR signal transduction. Overall, this Research Topic provides a timely overview of the many ways in which ligands can affect GPCR function and how this information can be used to develop novel therapeutics against various GPCR targets.

## Author contributions

PM: Writing – original draft, Writing – review & editing. MS: Writing – review & editing. CJ: Writing – review & editing. AP: Writing – review & editing. DH: Writing – review & editing, Writing – original draft.
